# Bioclimatic Thresholds, Thermal Constants and Survival of Mealybug, *Phenacoccus* *solenopsis* (Hemiptera: Pseudococcidae) in Response to Constant Temperatures on Hibiscus

**DOI:** 10.1371/journal.pone.0075636

**Published:** 2013-09-25

**Authors:** Gudapati Sreedevi, Yenumula Gerard Prasad, Mathyam Prabhakar, Gubbala Ramachandra Rao, Sengottaiyan Vennila, Bandi Venkateswarlu

**Affiliations:** 1 Division of Crop Sciences, Central Research Institute for Dryland Agriculture, Santoshnagar, Hyderabad, Andhra Pradesh, India; 2 National Centre for Integrated Pest Management, Pusa, New Delhi, India; Federal University of Viçosa, Brazil

## Abstract

Temperature-driven development and survival rates of the mealybug, *Phenacoccussolenopsis* Tinsley (Hemiptera: Pseudococcidae) were examined at nine constant temperatures (15, 20, 25, 27, 30, 32, 35 and 40°C) on hibiscus ( *Hibiscus* *rosa* *-sinensis* L.). Crawlers successfully completed development to adult stage between 15 and 35°C, although their survival was affected at low temperatures. Two linear and four nonlinear models were fitted to describe developmental rates of *P* *. solenopsis* as a function of temperature, and for estimating thermal constants and bioclimatic thresholds (lower, optimum and upper temperature thresholds for development: T_min_, T_opt_ and T_max_, respectively). Estimated thresholds between the two linear models were statistically similar. Ikemoto and Takai’s linear model permitted testing the equivalence of lower developmental thresholds for life stages of *P* *. solenopsis* reared on two hosts, hibiscus and cotton. Thermal constants required for completion of cumulative development of female and male nymphs and for the whole generation were significantly lower on hibiscus (222.2, 237.0, 308.6 degree-days, respectively) compared to cotton. Three nonlinear models performed better in describing the developmental rate for immature instars and cumulative life stages of female and male and for generation based on goodness-of-fit criteria. The simplified β type distribution function estimated T_opt_ values closer to the observed maximum rates. Thermodynamic SSI model indicated no significant differences in the intrinsic optimum temperature estimates for different geographical populations of *P* *. solenopsis* . The estimated bioclimatic thresholds and the observed survival rates of *P* *. solenopsis* indicate the species to be high-temperature adaptive, and explained the field abundance of *P* *. solenopsis* on its host plants.

## Introduction

Mealybugs (Hemiptera: Pseudococcidae) are important pests worldwide causing economic damage to several crops. The *Solenopsis* mealybug, *Phenacoccussolenopsis* Tinsley, first described from New Mexico, USA [1] was reported to infest cotton (*Gossypium hirsutum* L.) and 29 other hosts in Texas, USA [2]. This invasive species currently has a worldwide distribution spanning Central America, the Caribbean and Ecuador [3], Brazil [4], Pakistan and India [5], China [6], Nigeria [7] and Australia [8]. *P* *. solenopsis* is highly polyphagous with a record of 194 host plants [9]. *Hibiscus* *rosa* *-sinensis* L. is an evergreen herbaceous ornamental and landscape shrub, native to China and widely distributed in the tropics and sub-tropics. In the US, hibiscus is a popular flowering plant maintained in greenhouses all over the country [10]. In India, it is a common ornamental plant in the backyards and gardens nurtured for its bright and large flowers as religious offering. Hibiscus is a recorded host for *P* *. solenopsis* in Nigeria [7], China [6,11] and the Indian subcontinent [9,12]. As a perennial host, hibiscus appears to play an important role in the population dynamics of *P* *. solenopsis* on cotton during the crop season. After harvest of rainy season cotton by December, *P* *. solenopsis* survives mostly on alternate hosts such as *Abutilon* *indicum* (L.) and *Parthenium* *hysterophorus* L. [9]. As these weeds dry up in the hot summer during April-May in the rainfed tracts, *P* *. solenopsis* is mostly found surviving on *H* *. rosa* *-sinensis*. With the onset of monsoon rains in June, *P* *. solenopsis* population spreads to adjacent weeds that spring up early, and subsequently appears in adjoining fields planted to cotton [13]. Since *H* *. rosa* *-sinensis* is a popular and economically important ornamental species worldwide, and it serves as a host for *P* *. solenopsis* during summer time in the Indian subcontinent, the present study on development and survival on hibiscus gains significance.

Temperature is one of the major environmental factors influencing insects. Development, survival, adult longevity and fecundity data are vital for understanding the population dynamics of any insect species on a particular host [14]. Development of the polyphagous mealybug species, *Maconellicoccus* *hirsutus* (Green) at the same temperature was different when reared on five different hosts [15]. Understanding phenology of an insect species at different temperatures is crucial for predicting its seasonal occurrence and planning for integrated management. Many mathematical models describe insect developmental rate as a function of temperature [16-19]. Linear model [20] is widely used to explain the straight line relationship between the developmental rate and temperature in the limited range (15-30°C) and calculate lower developmental thresholds and thermal constants required to complete development of life stages. To describe the developmental rate more realistically and over a wider temperature range, several nonlinear models have been applied [21,22] to provide estimated values for optimum and maximum temperatures for development. However, estimation of thermal constant cannot be achieved by nonlinear models. They usually give a good fit to most experimental data, and many of them incorporate physiological and biochemical constants [16,17,21]. The biophysical explanation for the temperature-development relationship is that enzymes catalyze reactions such as those responsible for development within organisms. Exposure to extreme temperatures affects rates of enzyme activity by changing their conformation, and in some cases denaturing the proteins that regulate the biophysical processes of development, thereby stunting developmental rates [23-25].

In this paper, we applied several mathematical functions to describe the developmental rate of *P* *. solenopsis* on *H* *. rosa* *-sinensis* at nine constant temperatures (15 to 40°C). Development of *P* *. solenopsis* on cotton was proportional to temperature in the linear portion of the developmental rate curve [26]. We hypothesized a similar relationship on hibiscus and fitted linear models to the developmental rate data in the temperature range of 15-32°C to calculate the lower thresholds and thermal summation values useful to predict timing and phenology of life cycle events of *P* *. solenopsis* . As developmental times were longer at the upper temperature (35°C), we applied nonlinear models to describe this relationship and estimate the optimum and upper threshold temperatures for development. Prolific development of *P* *. solenopsis* field populations on hibiscus in the hot summer indicated a higher survival at warmer temperatures. We compared our results on the developmental thresholds, thermal constants and survival of *P* *. solenopsis* on hibiscus with that on cotton in India [26], on pumpkin and hibiscus in China [11,27]. Better understanding of the life history parameters of *P* *. solenopsis* on its key off-season host, hibiscus could provide leads to improve its prediction on cotton during the cropping season.

## Materials and Methods

### Mealybug colony establishment

Initial cultures of *P* *. solenopsis* collected on cotton (RRS No. 777-792/11, Insect Identification Service, IARI, New Delhi) were maintained for three generations on twigs of *H* *. rosa* *-sinensis* (bloom - pinkish red, no spots or splashes with eye zone small and red; leaves - semi-glossy, ovate with serrated margins). Different stages of *P* *. solenopsis* required for various experiments were drawn from this culture maintained at 27 ± 1°C, 65±5% relative humidity (RH) and 12: 12 h (L:D) photoperiod. In all the experiments, leaves of *H* *. rosa* *-sinensis* were used as host tissue. Fully expanded healthy leaves were excised, wiped with wet tissue and petioles were individually wrapped around with wet adsorbent cotton and placed in petri dishes (9 cm diameter) prior to release of insects. Leaves were changed every 3 to 4 days at 15-30°C and daily at 32-40°C.

### Development and survival

Development and survival of *P* *. solenopsis* was assessed at 9 constant temperatures: 15, 18, 20, 25, 27, 30, 32, 35 and 40°C (±1°C). For each temperature experiment, 20 mated females (30-35 d old) were transferred individually onto hibiscus leaves in petri dishes, and placed in an environmental growth chamber (MLR 350H, Sanyo, Japan) set at the test temperatures, 65 ± 5% RH, and a photoperiod of 12: 12 h (L:D). Neonate crawlers emerging from an ovisac of a mated female were transferred to a freshly prepared hibiscus leaf in a separate petri dish (10 crawlers/dish) and returned to the set temperature in the growth chamber. Twenty such replicates were maintained for each temperature. Petri dishes were examined daily using a stereomicroscope for shed exuviae which marked the successful completion of the current instar duration. Sex of each nymph was determined at the time of the second molt. From this point onwards, developmental times of males and females were recorded separately in segregated petri dishes. Daily mortality was recorded and instar survival in each replicate was determined as the percentage of surviving individuals at the start of each instar for female nymphs. For males, survival was assessed as the percentage of winged males that emerged from puparia formed at the end of the second molt. Survival from crawler to adult emergence was calculated as the percentage of crawlers that survived to adult stage. The percentage of female adults was calculated as a secondary sex ratio estimate. Preoviposition period was assessed by individually pairing each newly emerged female (<2 d) with 1-2 adult males developed at the same temperature.

### Linear models

Two models were evaluated to estimate the linear relationship between ecologically relevant temperatures and the rate of development of *P* *. solenopsis* . The first was the thermal summation model [20] which is given by the expression:

r(T)=a+bT (1)

where, *r* is the rate of development (=1/ Development time (*D*) in days), *T* is ambient temperature ( ^o^C); intercept (*a*) and slope (*b*) are the model parameters. Thermal constant, *k* (= 1/*b*), is the number of degree-days (DDs) or heat units above the threshold needed for completion of an instar (Table S1). Lower temperature threshold (T_min_) was determined as the x-intercept (= - *a*/*b*) which is the estimated lower temperature at which the rate of development is either zero or no measurable development occurs. Standard error (SE) values of T_min_ and *k* were calculated as described by Campbell [20].

The second linear model by Ikemoto and Takai [28] is given as:

$$DT=k+T_{min}D$$ (2)

where, *DT* is the product of the duration of development, *D* (days), and temperature, *T* ( ^o^C), *k* is thermal constant and T_min_ is the lower developmental threshold.

### Nonlinear models

Three empirical nonlinear models were fitted to the instar specific developmental rate data to estimate the optimum temperature threshold (T_opt_) and upper temperature threshold (T_max_). T_opt_ is the threshold temperature at which developmental rate is maximal, while T_max_ is the lethal threshold at which development ceases. Lactin-2 model [21], Briere-1 model [22] and simplified β type distribution function [29] were applied to assess the nonlinear relationship. In addition, a thermodynamic model referred to as the Sharpe-Schoolfield-Ikemoto model (SSI model) was used to estimate intrinsic optimum temperature for *P* *. solenopsis* [30]. All the nonlinear models described the relationship between developmental rate (1/D) and temperature (T).

The Lactin-2 model [21] is given by the expression:

$$\frac{1}{D}=e^{\rho \times T}-e^{\left[\rho \times T_{max}-\left(\left(T_{max}-T\right)÷\Delta \right)\right]}+\lambda$$ (3)

where, *D* is the mean development duration in days, *ρ* is the composite value for critical enzyme-catalyzed biochemical reactions as T increases to T_opt_, Δ is the difference between T_opt_ and T_max_ when thermal breakdown becomes the overriding influence and λ is a fitted coefficient that forces the nonlinear curve to intersect the *x*-axis and allows the estimation of lower developmental threshold. Although T_max_ is a parameter in the Lactin-2 model, it does not actually represent the upper temperature at which growth rate equals to zero (the upper developmental threshold). The true developmental threshold predicted by the model can be obtained only by simulation [31]. Thus both T_min_ and T_max_ were numerical approximations obtained as the roots of the fitted model by running the Newton-Raphson algorithm (SAS 9.2). Similarly, T_opt_ was obtained from the Lactin-2 equation by iterating the temperature parameter until the developmental rate was maximized [31].

The Briere-1 model [22] is given by the expression:

$$r\left(T\right)=aT(T-T_{min})\sqrt{\left(T_{max}-T\right)}$$ (4)

where, *r* is the developmental rate as a function of temperature (T), and ‘*a*’ is an empirical constant.

The simplified β type distribution function [29] fitted to the data is given by the expression:

$$\frac{1}{D}=k\left(\alpha -\frac{T}{10}\right){\left(\frac{T}{10}\right)}^{\beta }$$ (5)

where *k*, *α* and *β* are model parameters estimated by Marquardt’s nonlinear method. In this model, T_max_ and T_opt_ could not be derived directly from the equation as parameters. T_max_ was estimated graphically from the rapid decline of the right descending branch [29] and T_opt_ was derived using optimization [26].

The nonlinear thermodynamic SSI model [23-25] is given by the expression:

$$r\left(T\right)=\frac{\rho_{\text{φ}}\frac{T}{T_{\text{φ}}}exp\left[\frac{\Delta H_{A}}{R}\left(\frac{1}{T_{\text{φ}}}-\frac{1}{T}\right)\right]}{1+exp\left[\frac{\Delta H_{L}}{R}\left(\frac{1}{T_{L}}-\frac{1}{T}\right)\right]+exp\left[\frac{\Delta H_{H}}{R}\left(\frac{1}{T_{H}}-\frac{1}{T}\right)\right]}$$ (6)

where, developmental rate (r) is a function of temperature, *T* (in absolute temperature, K) (273.15K = 0°C), *R* is the gas constant (1.987 cal/deg/mol), *ΔH*_*A*_ is the enthalpy of activation of the reaction that is catalyzed by the enzyme (cal/mol), *ΔH*_*L*_ is the change in enthalpy associated with low-temperature inactivation of the enzyme (cal/mol), *ΔH*_*H*_ is the change in enthalpy associated with high-temperature inactivation of the enzyme (cal/mol), *T*_*L*_ is the temperature at which the enzyme is half active and half low-temperature inactive (K), *T*_*H*_ is the temperature at which the enzyme is half active and half high-temperature inactive (K), *T*_*ϕ*_ is the intrinsic optimum temperature at which the probability of enzyme being in the active state is maximal (K), and *ρ*_*ϕ*_ is the mean development rate at the intrinsic optimum temperature (*T_ϕ_*) assuming no enzyme inactivation (day^-1^).

### Statistical analysis

Generalized linear model with logit link function was used to model survival data with temperature as a fixed factor. The developmental duration data was checked for normality and found to be positively skewed. Cramer-von Mises and Anderson-Darling goodness-of-fit tests confirmed that the developmental duration data followed the gamma distribution. PROC GLIMMIX was applied to the nested design by assuming temperature as a fixed effect, and female-Id and crawlers nested within female-Id as random effects. Further, mean survival and development durations were separated using the Tukey-Kramer HSD (honestly significant difference) test at 5% level of significance.

Regression (Equation 1) was performed with replicate data using PROC REG to determine linear relationship between developmental rate (1/D) and temperature (*T*) to obtain *a* and *b* as regression parameters. Also the product of duration (*D*) and temperature (*T*) was regressed (Equation 2) on duration (D) to obtain T_min_ and *k* as the regression parameters. The two linear models were tested for significant differences in the estimates of T_min_ and thermal constants for different life stages by nonparametric Wilcoxon Signed Rank test.

PROC NLIN was performed to estimate parameters of nonlinear equation 3 (λ, *ρ*, T_max_ and Δ) and equation 4 (*a*, T_min_ and T_max_). PROC OPTMODEL was used to estimate the T_opt_ of the fitted models. Similarly, parameters of equation 5 (k, *α* and *β*) were estimated using PROC NLIN [32] and were used to derive T_opt_. The SSI model parameters in equation 6 were estimated by using the OptimSSI function in SSI package version 2.7 in R software using the default option of setting lower developmental threshold as the initial value of *T*_*L*_ (optT_L_ = 1). Confidence intervals (95%) for *T*_*ϕ*_ were estimated by the modified accelerated and bias corrected bootstrap method [30]. Model performance evaluation of the four nonlinear models was made based on goodness-of-fit statistics: Akaike information criterion (AIC), adjusted *R* ^*2*^ and root mean square error (RMSE). To compare developmental thresholds, thermal constants and survival among geographical populations of *P* *. solenopsis* , we extracted mean developmental data from published data sets [11,27]. Each author applied a different model to development duration data making it difficult to compare parameter estimates of different populations. Therefore, we adopted equation 6 to recalculate the lower developmental threshold (LDT, same as T_min_) and sum of effective temperature (SET, same as *k*) for different populations for a meaningful comparison. In one case, we could obtain replicate data sets of development duration at different temperatures from our previous study on cotton from the same laboratory [26] and hence it was possible to test the equality of LDTs and SETs for the local Warangal population on the two hosts: hibiscus in this study with those on cotton using equation 2 through analysis of covariance (ANCOVA) [33-35].

Pair-wise comparison of intrinsic optimum temperatures estimated from equation 6 for the same life stage between two geographical populations (data sets from [11,27]) or the local Warangal population on two hosts (this study and data set from [26]) was tested by calculating the 95% confidence interval (CI) of the difference between two groups of bootstrap replications of *T*_*ϕ*_ [30]. If the interval of difference contained 0, intrinsic optimum temperatures (*T*_*ϕ*_) of the first and second population were not statistically different [25].

To know how well the estimated bioclimatic thresholds explained the field abundance of *P* *. solenopsis* , we monitored the field incidence of the mealybug regularly on cotton during the crop season and on other hosts during off-season at identified locations in the south-central (18-22° N) and in the northern belt (28-32° N). Daily air temperatures were collected from the nearest meteorological observatory.

## Results

### Survival

*Phenacoccussolenopsis* successfully completed its development from crawler to adult emergence at all the temperatures between 15-35°C (Table 1). At 40°C, only eggs were produced which did not hatch. Crawlers were the most susceptible to temperature extremes while second and third instar females exhibited a high degree of survival. In case of male nymphs, no mortality was observed and adult males emerged from puparia at all temperatures (15-35°C). Overall, survival of crawlers to adult emergence was at its lowest (10.2%) at 15°C and at its highest (72.6%) at 32°C. Further, sex ratio in terms of proportion of females was >70% with higher female bias in the lower temperature range.

**Table 1:** Mean percent survival (± SEM) of different life stages of *P* *. solenopsis* females on hibiscus at constant temperatures and proportion of females.

Temperature (± 1°C)	Crawler	II Instar	III Instar	Crawler to adult	Proportion of females (%)
15	42.5 ± 1.0c	57.7 ± 3.3b	46.7 ± 3.3b	10.2 ± 0.1c	-*
18	55.0 ± 1.2bc	87.8 ± 2.5a	90.6 ± 2.4a	42.5 ± 1.6b	94.5 ± 2.2a
20	62.5 ± 1.2bc	84.2 ± 2.1a	91.6 ± 2.5a	45.4 ± 1.8ab	85.2 ± 4.3ab
25	69.5 ± 1.1abc	88.4 ± 2.6a	88.6 ± 2.4a	50.7 ± 2.1ab	78.7 ± 3.4ab
27	72.0 ± 1.2ab	89.2 ± 2.4a	89.1 ± 2.5a	52.5 ± 1.4ab	74.8 ± 2.9b
30	79.5 ± 0.5ab	87.5 ± 1.7a	94.3 ± 2.0a	61.0 ± 1.3ab	70.2 ± 0.8b
32	86.5 ± 1.3a	93.4 ± 1.7a	95.2 ± 1.7a	72.6 ± 1.3a	71.0 ± 2.6b
35	70.0 ± 1.3ab	90.5 ± 2.0a	92.8 ± 2.7a	54.7 ± 1.8ab	78.6 ± 1.5ab
F	15.98	7.73	9.81	18.51	3.82
df	7, 152	7, 152	7, 152	7, 152	6, 133
*P*	<0.0001	<0.0001	<0.0001	<0.0001	0.0015

Means within a column followed by the same letters are not significantly different at α = 0.05 (Tukey-Kramer HSD test).

* Low survival, hence not calculated.

### Development

Temperature significantly influenced the development of *P* *. solenopsis* nymphal instars and their cumulative development to adulthood (Table 2). However, maternal influence was not significant when included as a random effect in the analysis. In the temperature range of 15-32°C, developmental duration decreased with increase in temperature for both female and male nymphs. Time spent in each instar was the longest at 15°C and shortest at 32°C. Fastest developmental times were recorded for the second instar in females and pupal instar in males. Developmental durations of all nymphal instars increased above 32°C except for male prepupa. Cumulative development of female and male nymphs decreased from 57.5 and 61.5 d at 15°C to 10 and 12 d at 32°C, respectively. Duration of males was longer than females at all temperatures. Preoviposition period decreased from 35 d at 15°C to < 7 d at 30-32°C.

**Table 2:** Mean durations (d ± SEM) of *P* *. solenopsis* nymphal instars, their cumulative preimaginal development, and adult preoviposition reared on hibiscus at constant temperatures.

Temperature (± 1°C)	I instar	Female		Male			Cumulative		Preoviposition
		II instar	III instar	II instar	Prepupa	Pupa	Female	Male	
15	20.82 ± 0.14a	17.82 ± 0.16a	18.85 ± 0.22a	17.50 ± 0.50a	15.50 ± 0.65a	7.75 ± 0.48a	57.48 ± 0.38a	61.57 ± 1.34a	35.0 ± 0.6a
18	14.48 ± 0.04b	10.80 ± 0.07b	12.26 ± 0.12b	10.60 ± 0.24b	12.40 ± 0.24b	6.40 ± 0.24a	37.54 ± 0.14b	43.88 ± 0.49b	-
20	11.38 ± 0.03c	9.00 ± 0.06c	10.06 ± 0.08c	8.10 ± 0.07c	8.53 ± 0.15c	4.23 ± 0.13b	30.43 ± 0.12c	32.25 ± 0.16c	21.2 ± 0.8b
25	6.55 ± 0.03d	5.42 ± 0.04d	6.72 ± 0.06d	5.04 ± 0.03d	5.57 ± 0.12d	3.46 ± 0.16bc	18.69 ± 0.09d	20.59 ± 0.15d	9.9 ± 0.3c
27	5.45 ± 0.03e	4.58 ± 0.03e	5.33 ± 0.07e	4.24 ± 0.07e	4.64 ± 0.08e	3.01 ± 0.11bcd	15.35 ± 0.09e	17.32 ± 0.15e	8.8 ± 0.4c
30	4.14 ± 0.04f	3.48 ± 0.06f	4.81 ± 0.03f	3.13 ± 0.06f	3.78 ± 0.08f	2.58 ± 0.08cde	12.43 ± 0.07f	13.62 ± 0.13f	6.8 ± 0.1d
32	3.01 ± 0.03h	2.97 ± 0.03h	3.89 ± 0.04h	2.95 ± 0.05f	3.68 ± 0.06f	2.16 ± 0.05e	9.87 ± 0.06h	11.79 ± 0.08h	6.0 ± 0.1e
35	3.70 ± 0.04g	3.25 ± 0.03g	4.35 ± 0.03g	3.15 ± 0.09f	3.53 ± 0.11f	2.23 ± 0.13de	11.29 ± 0.06g	12.60 ± 0.18g	6.25 ± 0.2de
F	9584.00	4636.06	3633.02	595.44	335.93	59.79	14860.10	1896.24	262.45
df	7, 152	7, 152	7, 104	7, 104	7, 104	7, 104	7, 152	7, 104	6, 95
*P*	<0.0001	<0.0001	<0.0001	<.0001	<0.0001	<0.0001	<0.0001	<0.0001	<0.0001

Means within a column followed by the same letter are not significantly different at α = 0.05 (Tukey-Kramer HSD test); - not recorded

### Developmental thresholds and thermal constants

The linear relationship between developmental rate and temperatures in the range of 15 to 32°C for female and male instars and their cumulative development was explained well by both the linear models (*R* ^2^ > 0.91, *P* < 0.0001) except for male pupa (Table 3). However, the linear relationship was much stronger for the combined instars of male prepupa and pupa (data not shown, *R* ^2^ = 0.94, *P* < 0.0001). Equation 2 gave consistently narrower estimates of standard errors of T_min_ for all the instars. Thermal constants (k) estimated from equation 1 were 222.2 and 237.0 DDs compared to 230.4 and 246.7 DDs from equation 2 for cumulative female and cumulative male, respectively. Thermal constants for generation estimated by the two linear models were close (308.6 and 306.3 DDs). Wilcoxon signed rank test revealed that T_min_ (S = 14.5, *P* = 0.1602) and SET (S=16.5, *P* = 0.1055) for all individual nymphal instars and their cumulative life stages obtained from linear equation 1 were not significantly different from those obtained from linear equation 2. ANCOVA test confirmed rate isomorphy as the interaction term (development duration × host) was not significant (*P* > 0.05) for cumulative female (*F* = 3.51, *P* = 0.06), cumulative male (*F* = 0.17, *P* = 0.68) and for generation (*F* = 0.02, *P* = 0.89) (Table 4). Thus, there was no significant difference in the slopes of equation 2 corresponding to the two hosts, hibiscus and cotton. However, ANCOVA without the interaction term, gave *p*-values of slopes <0.05. Hence, the thermal constants for these stages of the local population were significantly lower on hibiscus compared to cotton.

**Table 3:** Lower temperature threshold (T_min_) and thermal constant (k) estimates for life stages of *P* *. solenopsis* on hibiscus from linear models at selected constant temperature ranges^**a**^ *.*

Life stage	Equation	Temperature range (°C)	Linear regression^a^	R^2^	df	F	*P*	T_min_ (°C) ± SE	*k* (DD) ± SE
Crawler	1	18-30	r(T) = -0.193 ± 0.014T	0.980	1, 98	4814.0	<0.0001	13.63 ± 0.16	70.5 ± 1.02
	2	18-30	DT = 74.48 ± 13.12D	0.994	1, 98	16310.5	<0.0001	13.12 ± 0.10	74.5 ± 0.95
Female II instar	1	18-30	r(T) = -0.206 ± 0.016T	0.961	1, 98	2410.0	<0.0001	12.83 ± 0.24	62.3 ± 1.27
	2	18-30	DT = 65.72 ± 12.30D	0.984	1, 98	5924.02	<0.0001	12.30 ± 0.10	65.7 ± 0.95
Female III instar	1	18-32	r(T) = -0.141 ± 0.012T	0.964	1, 118	3195.8	<0.0001	11.70 ± 0.26	83.0 ± 1.47
	2	18-32	DT = 85.73 ± 11.33D	0.975	1, 118	4656.9	<0.0001	11.33 ± 0.17	85.7 ± 1.29
Male II instar	1	18-32	r(T) = -0.259 ± 0.019T	0.937	1, 86	1272.8	<0.0001	13.80 ± 0.39	53.1 ± 1.49
	2	18-32	DT = 55.70 ± 13.23D	0.976	1, 86	3537.7	<0.0001	13.23 ± 0.22	55.7 ± 1.14
Prepupa	1	18-30	r(T) = -0.192 ± 0.015T	0.916	1, 67	728.4	<0.0001	12.65 ± 0.51	65.9 ± 2.44
	2	18-30	DT = 65.63 ± 12.76D	0.972	1, 67	2288.4	<0.0001	12.76 ± 0.27	65.6 ± 1.66
Pupa	1	18-32	r(T) = -0.189 ± 0.020T	0.736	1, 86	239.9	<0.0001	9.50 ± 1.17	50.2 ± 3.25
	2	18-32	DT = 43.04 ± 12.08D	0.777	1, 86	299.8	<0.0001	12.08 ± 0.70	43.0 ± 2.32
Cumulative male nymph	1	18-30	r(T) = -0.055 ± 0.004T	0.974	1, 67	2488.1	<0.0001	13.00 ± 0.27	237.0 ± 4.75
	2	18-30	DT = 246.75 ± 12.50D	0.990	1, 67	6466.7	<0.0001	12.50 ± 0.16	246.7 ± 3.55
Cumulative female nymph	1	18-30	r(T) = -0.056 ± 0.005T	0.988	1, 98	8218.6	<0.0001	12.50 ± 0.14	222.2 ± 2.45
	2	18-30	DT = 230.45 ± 12.12D	0.993	1, 98	13635.2	<0.0001	12.12 ± 0.10	230.4 ± 2.57
Preoviposition	1	15-32	r(T) = -0.135 ± 0.008T	0.946	1, 88	1580.9	<0.0001	14.30 ± 0.47	105.9 ± 3.73
	2	15-32	DT = 110.45 ± 13.98D	0.956	1, 88	1916.4	<0.0001	13.98 ± 0.24	110.5 ± 2.82
Generation (crawler to crawler)	1	20-30	r(T) = -0.046 ± 0.003T	0.975	1, 64	2504.5	<0.0001	14.07 ± 0.24	308.6 ± 6.17
	2	20-30	DT = 306.30 ± 14.16D	0.991	1, 64	6715.8	<0.0001	14.16 ± 0.17	306.3 ± 5.85

a rate (r) as a function of temperature T, in equation 1; DT is product of development duration (D) and temperature (T) in equation 2

**Table 4:** Results of ANCOVA for testing equivalence of lower developmental threshold (T_min_) estimates for the local population of *P* *. solenopsis* reared on hibiscus (this study) and cotton [26].

Model	Source of variation	df	Cumulative female		Cumulative male		Generation	
			F	*P*	F	*P*	F	*P*
ANCOVA with interaction term	Duration	1	7061.11	<0.0001	3551.9	<0.0001	2789.87	<0.0001
	Host	1	145.71	<0.0001	47.88	<0.0001	29.13	<0.0001
	Duration × host	1	3.51	0.0624	0.17	0.6802	0.02	0.894
ANCOVA without interaction term	Duration	1	7435.11	<0.0001	3758.56	<0.0001	4557.94	<0.0001
	Host	1	721.82	<0.0001	587.88	<0.0001	190.64	<0.0001

**Figure 1:**
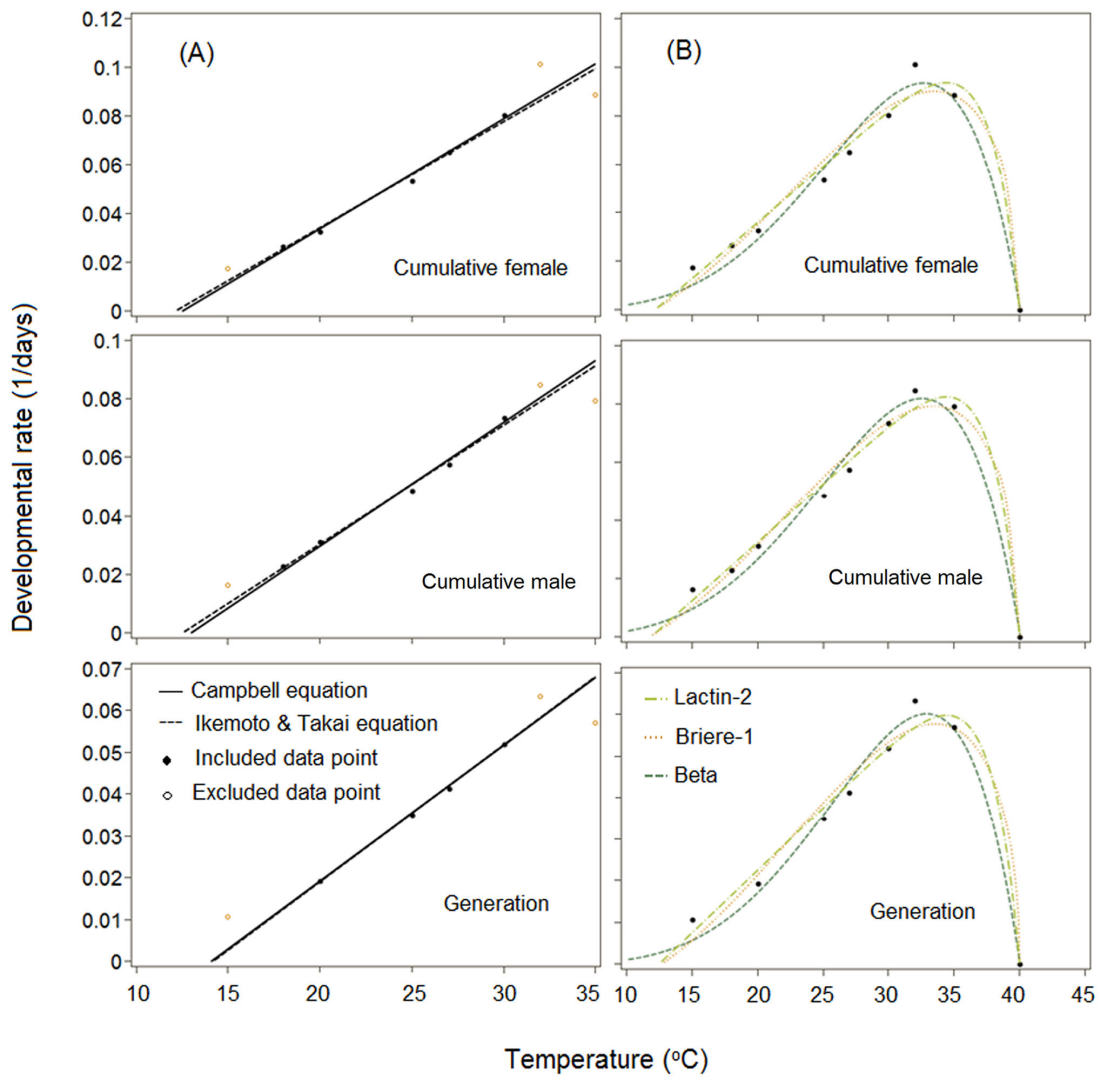
Linear fit (A) and nonlinear fit (B) to mean developmental rate data for cumulative preimaginal development and generation of the mealybug *Phenacoccussolenopsis* .

**Figure 2:**
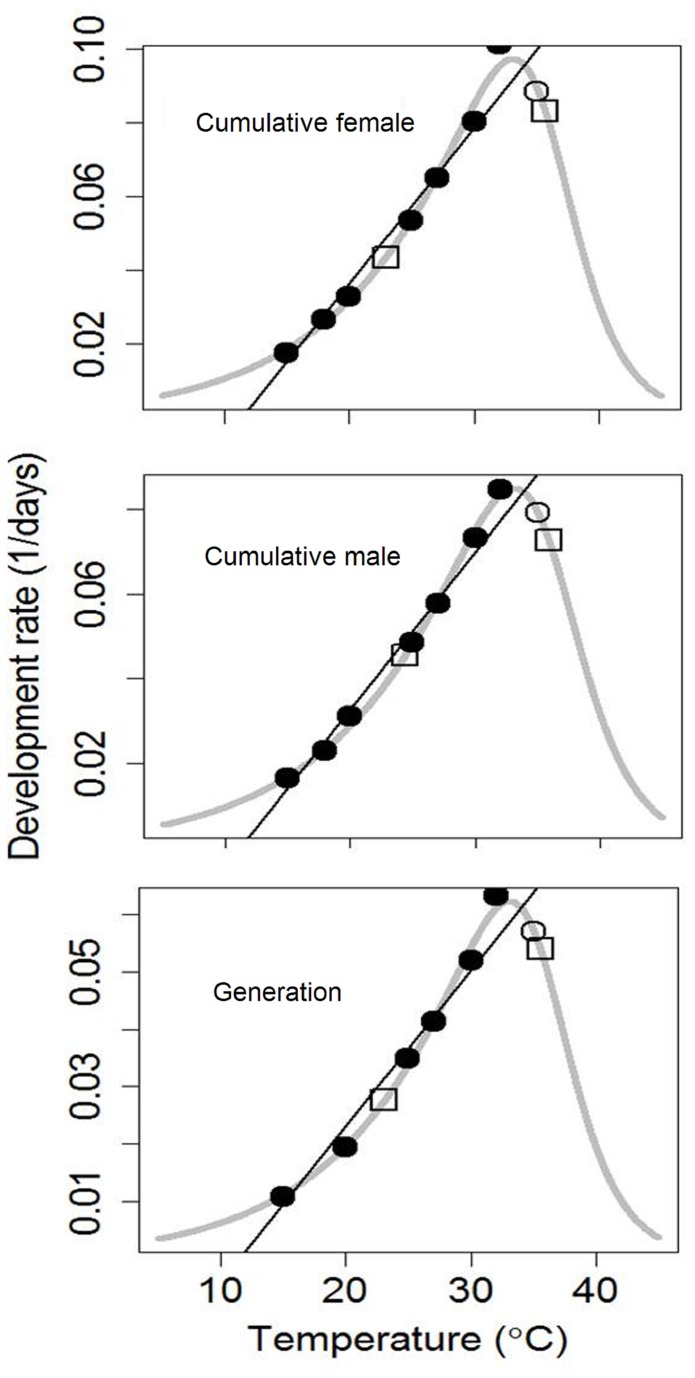
Comparison between the observed and theoretical values of the temperature-dependent development rates of the mealybug *Phenacoccussolenopsis* : the grey curve shows the predicted values by SSI model; the dark solid line shows predictions from the Ikemoto and Takai linear model; the open and closed circles are observed values used in the nonlinear fitting. The closed circles are observed values used in the linear fitting. Open squares from left to right represent the developmental rates at *T*_*ϕ*_ and *T*_*H*_.

The nonlinear relationship between developmental rate and temperature for different life stages of *P* *. solenopsis* on hibiscus was fitted well by all the four nonlinear models viz., Lactin-2, Briere-1 and β type distribution function and SSI model by additionally including rates at 35 and 40°C (Figures 1 and 2, Table 5). T_min_ estimated using nonlinear Briere-1 and Lactin-2 models for cumulative female were close to the linear model estimates, but the standard error values of T_min_ provided by Briere-1 model were higher. Optimum temperature (T_opt_) thresholds obtained were in the range of 33.9 to 34.6°C from Lactin-2 model, while they were 32.9 to 33.9°C with Briere-1 and 31.7 to 33.1°C with the simplified β type distribution function. SSI model estimated the intrinsic optimum temperature (*T*_*ϕ*_) between 21.0-24.5°C for all instars except the first (16.2°C). Empirical nonlinear models estimated the upper developmental threshold (T_max_) between 40 and 40.5°C for all the individual nymphal instars and for their cumulative development. SSI model estimates of *T*_*H*_ were in the range of 35.1 to 38.6°C. Higher values of adjusted *R* ^*2*^ (0.976 to 0.992), lower RMSE (0.0094 to 0.0221) and lower AIC (-81.3 to -65.9) were obtained with Lactin-2 model for the male immature instars and the third instar female (Table 5) indicating a better fit compared to the other nonlinear models. Lactin-2 and β type distribution function performed better in case of cumulative female (adjusted *R* ^2^ = 0.96, RMSE = 0.007 and AIC range = -86.7 to -87.0). In case of the cumulative male, both SSI and Lactin-2 models performed better while for generation, beta type distribution function performed better based on the goodness-of-fit criteria.

**Table 5:** Estimates of nonlinear model parameters and temperature thresholds (^**o**^C) for development of life stages of *P* *. solenopsis* on hibiscus at full range of constant temperatures.

Nonlinear model	Parameter/ Model fit statistic		Female		Male			Cumulative		Generation (crawler to crawler)
		I instar	II instar	III instar	II instar	Prepupa	Pupa	Female	Male	
Lactin-2	T_min_	13.0	12.0	10.6	12.1	10.7	7.3	12.2	11.9	12.5
	T_opt_	34.6	34.3	34.0	33.9	34.2	34.6	34.4	34.4	34.5
	T_max_	40.0	40.0	40.0	40.5	40.0	40.0	40.0	40.0	40.0
	RMSE × 10^2^	3.23	1.91	1.34	1.70	0.94	2.21	0.70	0.43	0.44
	Adj. R^2^	0.917	0.975	0.976	0.981	0.992	0.980	0.960	0.980	0.963
	AIC	-59.10	-68.56	-74.88	-70.61	-81.28	-65.92	-86.69	-95.49	-84.30
Briere-1	T_min_	13.4 ± 1.79	12.3 ± 1.25	10.3 ± 1.36	12.0 ± 1.02	10.8 ± 0.94	8.2 ± 1.97	12.1 ± 1.44	11.8 ± 1.16	12.7 ± 1.52
	T_opt_	33.7	33.5	33.2	33.5	33.3	32.9	33.5	33.4	33.9
	T_max_ ± (SE × 10^3^)	40.0 ± 0.2	40.0 ± 0.1	40.0 ± 0.1	40.0 ± 0.1	40.0 ± 0.1	40.0 ± 0.1	40.0 ± 0.1	40.0 ± 0.1	40.0 ± 0.1
	RMSE × 10^2^	3.28	2.24	1.50	1.86	1.28	3.28	0.74	0.50	0.45
	Adj. R^2^	0.914	0.965	0.970	0.978	0.985	0.956	0.955	0.972	0.961
	AIC	-59.14	-66.06	-73.24	-69.35	-76.12	-59.15	-86.04	-92.97	-84.14
Beta type	T_opt_	33.1	32.7	32.1	32.5	32.2	31.7	32.7	32.5	32.9
	T_max_	40.0	40.0	40.1	40.0	40.1	40.1	40.0	40.1	40.0
	RMSE × 10^2^	2.68	2.10	1.86	1.75	1.89	4.93	0.70	0.53	0.36
	Adj. R^2^	0.943	0.969	0.954	0.980	0.966	0.90	0.960	0.969	0.975
	AIC	-62.78	-67.18	-69.33	-70.43	-69.09	-51.83	-87.01	-91.90	-87.78
SSI	*T*_*ϕ*_	16.2	23.0	24.0	22.6	24.5	21.0	22.9	24.4	23.1
	RMSE × 10^2^	2.16	1.46	2.34	1.77	1.78	5.04	0.79	0.32	0.34
	Adj. R^2^	0.956	0.981	0.901	0.974	0.959	0.840	0.935	0.985	0.969
	AIC	-63.97	-70.23	-62.75	-67.19	-67.09	-50.45	-80.17	-94.46	-79.12

Metaanalysis of mean development data by applying SSI model to the published data sets yielded estimates of intrinsic optimum temperature for different geographical populations of *P* *. solenopsis* . *T*_*ϕ*_ estimates for cumulative development of the female nymphs for the local population was 22.9°C on hibiscus, 22.8°C on cotton; 23.0°C for the Guangzhou population on hibiscus, and 18.7°C for the Zhejiang population reared on pumpkin (Table 6). *T*_*ϕ*_ estimates were comparatively higher for cumulative development of male on all the hosts.

**Table 6:** Comparison of SSI model estimates among populations of *P* *. solenopsis* reared on different hosts^a^.

S. No.	Population	Host plant	Constant temperatures ^b^	Parameter	Estimates (°C)			Reference
					Cumulative female	Cumulative male	Generation (crawler to crawler)	
1	Warangal, India 18°N, 79°E	Hibiscus	♀: 18, 20, 25, 27, 30 (15, 32, 35); ♂:15, 18, 20, 25, 27, 30, 32 (35); Generation: 20, 25, 27, 30,(35)	LDT	12.1	12.32	14.0	This study
				SET	230.3	250.6	312.2	
				*T*_*ϕ*_ ** ^*c*^	22.9 (18.7, 26.5)	24.4 (17.1, 25.1)	23.1 (22.8, 23.4)	
2	Warangal, India 18°N, 79°E	Cotton	♀: 18, 20, 22, 25, 27, 30, 32 (36); ♂: 20, 25, 27, 30,(36); Generation: 20, 25, 30,(35)	LDT	11.55	12.36	12.36	[26]
				SET	322.7	318.8	482.8	
				*T*_*ϕ*_ ** ^*c*^	22.8 (20.7, 26.5)	23.7 (23.1, 25.0)	23.6 (23.5, 24.5)	
3	Guangzhou, China 23°N, 113°E	Hibiscus	18, 21, 24, 27, 30,(33)	LDT	13.31	13.11	13.34	[11]
				SET	170.1	222.7	370.8	
				*T*_*ϕ*_^*c*^	23.0 (22.6, 24.6)	23.3 (23.3, 25.0)	22.8 (22.5, 23.0)	
4	Zhejiang, China 30°N, 120°E	Pumpkin	18, 20, 24, 26, 28,(30)	LDT	8.54	9.88	8.32	[27]
				SET	526.8	428.4	808.5	
				*T*_*ϕ*_	18.7 (18.4, 18.7)	19.1 (18.7, 21.5)	18.5 (18.5, 22.6)	

^a^ Recalculated with mean development duration datasets at constant temperatures from previous studies at S. No. 2, 3 & 4;

^b^ Temperatures (± 1°C) in the linear range for LDT and SET estimates, and additional temperatures in parentheses for nonlinear estimate of *T*_*ϕ*_;

^c^ 95% confidence intervals of *T*_*ϕ*_ estimated from the method of modified Accelerated and Bias Corrected bootstrap confidence intervals (mABCSSI method) [30].

## Discussion

### Survival and development

Temperature is the most important environmental factor that influences insect development and survival. Due to its invasive spread to distant places from its origin in USA and ability to survive on different host plants, many recent studies focused on the temperature dependent biology of *P* *. solenopsis* populations from different geographical areas. Constant temperature response experiments were used to estimate parameters of phenology models to bring out the thermal differences in development times or their inverse, development rates. However, these studies differed in host plants, constant temperature range and models used. Two studies adopted the widely used equation 1 [11,26], while another study [27] used the logistic model. Few other studies confined their results to application of ANOVA to the developmental duration data of life stages [36,37]. The geographical populations of *P* *. solenopsis* showed variation in development time, lower threshold and thermal constant. These differences can be attributed to multiple factors such as experimental conditions, host-plant quality and thermal adaptation to the geographical area [38]. Survival during the life stage is another factor measured in experiments designed to estimate development time using constant temperatures. The resultant observations of survival are typically modal with poorest survival at low and high temperatures [39]. The causes of reduced survival near threshold temperatures can include heat or cold injury as well as bottlenecks when discrete developmental events such as egg hatch or nymphal moult cannot occur. Among mealybug species, *P* *. solenopsis* could successfully complete its development at 15°C like the Madeira mealybug, *Phenacoccusmadeirensis* Green [40] but unlike *P* *. marginatus* which failed to complete development at this temperature [10] or eggs of *M* *. hirsutus* which failed to eclose [41]. However, the survival of *P* *. solenopsis* was greatly reduced at this lower temperature. Males could successfully emerge out of puparia at all the temperatures tested, as observed in another Phenacoccus species, *P* *. madeirensis* [40]. Overall survival of crawlers to adult stage was higher on hibiscus at 35°C (Table 1) compared to cotton [26].

Temperature had a pronounced effect on *P* *. solenopsis* reared on hibiscus with accelerated development to a maximum rate at 32°C, beyond which it decelerated and ceased at 40°C (Figures 1 and 2). In this study, the average cumulative developmental times for *P* *. solenopsis* female nymphs at 20 and 30°C on hibiscus were markedly faster compared to the development on cotton [26] and pumpkin [27] but were closer to those reported on the same host, hibiscus (this study and [11]). Preoviposition period of mated females in this study was 2-3 days shorter on hibiscus compared to cotton. Observed differences related to the geographical populations and hosts are discussed later on the basis of parameter estimates of linear and nonlinear models.

### Evaluation of models

A linear approximation of relationship between developmental rate and temperature gives the most appropriate fit within the quasi-linear range of temperatures [16]. Outside this range, developmental rate deviates from the straight line and hence some data points are to be excluded in the linear fit [17,28,35]. We selected the linear range of 18-30°C (Table 3) after taking into account higher R^2^ values, lower survival of crawlers to adulthood at 15°C, and rapid development of instars at 32°C. The data points at 15 and 32°C were excluded while fitting the longest developmental times of *P* *. solenopsis* i.e. cumulative female, cumulative male and generation due to low sample numbers at the lower temperature and use of daily observation data in this study even at the higher temperature [42]. Equation 1 is widely used for calculating the lower temperature thresholds [17-19] and it is the simplest method for estimation of thermal constant [43]. We employed equation 2 to increase precision of parameter estimates as demonstrated by Iketmoto and Takai [28]. Although, higher *R* ^*2*^ were obtained with equation 2, parameter estimates were statistically similar. Even though there was no apparent advantage with equation 2 over equation 1, the former allowed testing of developmental rate isomorphy for the local population of *P* *. solenopsis* on two hosts (Table 4).

All the nonlinear models described well the developmental rate curve for all the instars and their cumulative development (Figures 1 and 2, Table 5). The fact that the nonlinear models include developmental data from higher temperatures where the relationship is no longer linear is considered an advantage in estimating the theoretical T_opt_ and T_max_ [29]. The advantage of Lactin-2 over the simplified β type distribution function and Briere-1 model was the incorporation of parameters which have a biological interpretation. However, T_opt_ values from Lactin-2 model were slightly overestimated compared to the temperature at which the maximum developmental rate was observed. Simplified β type distribution function gave a more biological reason to prefer it to other models as T_opt_ values were in accordance with the fastest development rates observed at 32°C on hibiscus (Tables 2 and 5) and on cotton [26]. The estimates of *T*_*H*_ values from SSI model (Table S2) were lower than the upper lethal temperatures (T_max_). This was reflected in the higher survival of nymphal instars and the overall survival of crawlers to adulthood observed at 35°C (Table 1). *T*_*H*_ estimates indicated that the enzymes were half active and half inactive between 35.1 and 38.6°C and provided a physiological basis for the nonlinear development observed in different instars at the higher temperatures.

Mated females of *P* *. solenopsis* exhibit ovoviviparous reproduction as the crawlers are observed on the same day of egg deposition [26]. As a result, preimaginal development is synonymous with nymphal stage without a distinct egg stage unlike in *P* *. madeirensis* [40]. Therefore, we could not apply the rate isomorphy principle where the proportion of time spent in preimaginal developmental stages of a population does not change with temperature [44]. However, if rate isomorphy is common, there should be little variation in the lower developmental threshold between stages within species and populations [25,42] which was found true for the local population of *P* *. solenopsis* on two hosts, hibiscus and cotton. However, the local population required significantly lower thermal constants on hibiscus than on cotton. Shorter development times are indicative of better host suitability [15].

Variation in LDTs of the same developmental stage among different geographical populations suggest genetic capacity of a species to shift its thermal sensitivity for adapting to different climatic conditions. LDT tends to vary with the temperature of the niche to which the organism is adapted and there is a trade-off between LDT and SET among conspecifics [42,45]. In this study, we applied the SSI model to recalculate LDT and SET values, and estimated intrinsic optimum temperatures (*T*_*ϕ*_) from the mean developmental data of geographical populations which permitted statistical comparison of bioclimatic thresholds on different hosts. There were no significant differences in LDT and SET estimates between the Warangal population (this study) and the Guangzhou population [11], both reared on hibiscus (Table S3). However, these values differed significantly with that of Zhejiang population reared on pumpkin [27]. Undue prolongation of developmental times reflected in larger thermal constants for completing life stages suggested that pumpkin is not a suitable host for *P* *. solenopsis* . Thermal characteristics among different populations of species may vary with the food source [46]. Further studies may be required to test whether the significantly different LDT estimate of Zhejiang population of *P* *. solenopsis* on pumpkin is host induced or reflects a shift in thermal sensitivity to its niche field environment in the higher latitudes (30° N).

The most important parameter estimated by the nonlinear thermodynamic SSI model is the intrinsic optimum temperature (*T*_*ϕ*_) which is the temperature at which the probability of enzymes being in the active state is maximal [23]. It is considered as the most suitable temperature for an insect to exist and reflects its adaptation to the thermal environment. *T*_*ϕ*_ estimated from mean values of developmental rates at different temperatures means that the probability of the enzymes being active for the population is maximal and hence applies to the whole population. Also, *T*_*ϕ*_ might be constant for an insect species. This means that among different geographical populations there is presumably no significant difference in this parameter [25]. The pair-wise comparison of the intrinsic optimum temperatures of geographical populations revealed no differences for *T*_*ϕ*_ (Table S4), thus, substantiating constancy in *T*_*ϕ*_ among geographical populations of *P* *. solenopsis* . Intrinsic optimum temperatures of geographical populations of diamond back moth reared on different hosts did not differ and the peak crop damage coincided when the daily average air temperatures equalled or approximated *T*_*ϕ*_ [25].

Further, we applied the bioclimatic thresholds to explain the field abundance of *P* *. solenopsis* in the south-central cotton growing belt of India. In this tract, cotton is sown during June-July and harvested by December-January. Mean daily air temperatures in the optimum range for maximum development rate (T_opt_, 32-33°C) estimated from β type distribution function (Table 5) prevailed during summer (April-May). During this period, *P* *. solenopsis* was mostly recorded on its alternate host, *H* *. rosa* *-sinensis*. Mean daily air temperatures in the range of 95% CIs of intrinsic optimum temperature (*T*_ϕ,_ 18.7 to 26.5°C) estimated for cumulative female on hibiscus (this study) from the thermodynamic SSI model (Table 5) prevailed between October and February. Peak abundance of *P* *. solenopsis* was recorded on cotton in this window period (October to November). It is inferred that the average air temperatures closer to T_opt_ coincided with the abundance of the mealybug during the off-season on its alternate host, *H* *. rosa* *-sinensis*, while the average air temperatures within the 95% CI of intrinsic optimum temperature coincided with its abundance on its main host, cotton. Abundance of *P* *. solenopsis* on cotton is perhaps also aided by the withdrawal of pesticide sprays by farmers towards the crop maturity. The population subsequently shifted to the available alternate hosts after the termination of the cotton crop during December-January. Further, in this south-central cotton belt of India (18-22° N), daily minimum temperatures are >LDT (12.5°C for cumulative female, Table 3) in the coldest month of January and T_max_ (40°C) during the hottest month of May. Similarly, in the northern latitudes (Haryana and Punjab, 28-32° N), minimum temperatures fall below 5°C and daily mean air temperatures are closer to LDT during January, while daily maximum temperatures reach above 40°C during June. Therefore, estimated LDT and >T_max_ thresholds for *P* *. solenopsis* could possibly explain the wider annual population fluctuations in the northern belt [47]. However, there are factors other than temperature, such as nutrition, humidity, photoperiod and natural enemies that regulate population dynamics of a species.

Use of ecologically relevant constant temperatures and use of appropriate models in this study on hibiscus made it possible to compare the developmental thresholds of *P* *. solenopsis* with those estimated on cotton, its seasonal host [26]. For the local population, we found no significant difference in LDTs between the life stages reared on hibiscus and cotton. The estimates of T_opt_ and T_max_ from the empirical nonlinear models were similar on both the hosts and were closer to the observed rates of development and survival. However, cumulative preimaginal development of female and male nymphs, and generation were much faster on hibiscus and required lower thermal constants (DDs) for completion. Overall, higher values of bioclimatic thresholds (T_min_, T_opt_ and T_max_), lower thermal constants, higher survival rates and shorter preoviposition period at higher temperatures suggest that *P* *. solenopsis* exhibits the traits of a high-temperature adapted tropical species [45].

In this paper, we applied the thermodynamic SSI model to the published mean developmental duration data and found no significant differences in LDT and *T*_*ϕ*_ estimates for the geographical populations of *P* *. solenopsis* on different hosts with the exception of *P* *. solenopsis* population on pumpkin. *T*_*ϕ*_ estimates for *P* *. solenopsis* are within the usual range of predicted intrinsic optimum temperatures (18-23°C) based on development rate for some insects [23-25]. We made an attempt to make use of the estimated model parameters (T_opt_ and *T*_*ϕ*_) to understand the annual cycle of *P* *. solenopsis* and its seasonal phenology in the cotton agro-ecosystems of the south-central India. Bioclimatic parameter estimates from this study could be used to map the potential geographical distribution of *P* *. solenopsis* . These estimates along with other eco-climatic indices can be used as inputs for simulation modeling [47,48] or as empirical evidence for model validation. Such a method was applied for forecasting of *Helicoverpa* populations in Australia [49]. Our results suggest that *H* *. rosa* *-sinensis* is among the preferred off-season hosts for *P* *. solenopsis* and supports its development and survival at higher temperatures during summer. This implies that the population regulation of *Solenopsis* mealybug during summer by periodical monitoring, pruning and destroying of mealybug infested shoot apices on hibiscus is very crucial for its effective management to reduce infestation on cotton in the ensuing season. The world over, hibiscus is an important ornamental plant. Hence, the developmental data of *P* *. solenopsis* on hibiscus provided in this study would be useful for monitoring and planning control strategies on this plant and several other crops as well.

## Supporting Information

Table S1 **Definition of acronyms used.**
(DOCX)

Table S2 **Estimates of nonlinear model parameters fitted to the mean rates for development of life stages of *P* *. solenopsis* on hibiscus at constant temperatures.**
(DOCX)

Table S3 **Results of t-test for evaluation of lower development thresholds (LDTs) and sum of effective temperatures (SETs) of geographical populations of *P* *. solenopsis* .**
(DOCX)

Table S4 **Evaluation of intrinsic optimum temperature (*T*_*ϕ*_) estimates from SSI model for geographical populations of *P* *. solenopsis* reared on different hosts.**
(DOCX)
