# Supplementary material for: Bioclimatic Thresholds, Thermal Constants and Survival of Mealybug, Phenacoccus solenopsis (Hemiptera: Pseudococcidae) in Response to Constant Temperatures on Hibiscus
Source: PLoS One. 2013 Sep 25;8(9):e75636. doi: 10.1371/journal.pone.0075636 (PMC3783440; doi:10.1371/journal.pone.0075636)
Supplement: Table S1 — Definition of acronyms used. (DOCX) [file pone.0075636.s001.docx]

**Table S1: Definition of acronyms used**

| **Acronym** | **Full form** | **Definition** |
| --- | --- | --- |
| AIC | Akaike information criterion | Measure of the relative [goodness of fit](http://en.wikipedia.org/wiki/Goodness_of_fit) of a [statistical model](http://en.wikipedia.org/wiki/Statistical_model) |
| ANCOVA | Analysis of covariance | A [general linear model](http://en.wikipedia.org/wiki/General_linear_model) which blends [ANOVA](http://en.wikipedia.org/wiki/ANOVA) and [regression](http://en.wikipedia.org/wiki/Regression_analysis) and evaluates whether population means of a [dependent variable](http://en.wikipedia.org/wiki/Dependent_variable) are equal across levels of a categorical [independent variable](http://en.wikipedia.org/wiki/Independent_variable), while statistically controlling for the effects of other continuous variables that are not of primary interest, known as [covariates](http://en.wikipedia.org/wiki/Covariate) |
| DD | Degree days | The total amount of heat units required, between the lower and upper thresholds, for an organism to develop from one point to another in its life cycle |
| *k* | Thermal constant | Number of degree days or heat units above Tmin needed for completion of an instar |
| LDT | Lower temperature threshold | Temperature at which the rate of development is zero or no measurable development occurs |
| RMSE | Root mean square error | Measure of the differences between values predicted by a model and the values actually observed. |
| SET | Sum of effective temperatures | Same as thermal constant *k* |
| SSI model | Sharpe-Schoolfield model modified by Ikemoto | A nonlinear mathematical models for describing temperature dependent development rates |
| T_max_ | Upper temperature threshold | Temperature at which development ceases |
| T_min_ | Lower temperature threshold | Same as LDT |
| T_opt_ | Optimum temperature | Temperature at which developmental rate is maximum |
| T_ɸ_ | Intrinsic optimum temperature | Temperature at which the probability of enzyme being in active state is maximal |
| T_H_ | High temperature inactivation | Temperature at which the enzyme is half active and half high-temperature inactive |
| T_L_ | Low temperature inactivation | Temperature at which the enzyme is half active and half low-temperature inactive |
