# Supplementary material for: Bioclimatic Thresholds, Thermal Constants and Survival of Mealybug, Phenacoccus solenopsis (Hemiptera: Pseudococcidae) in Response to Constant Temperatures on Hibiscus
Source: PLoS One. 2013 Sep 25;8(9):e75636. doi: 10.1371/journal.pone.0075636 (PMC3783440; doi:10.1371/journal.pone.0075636)
Supplement: Table S2 — Estimates of nonlinear model parameters fitted to the mean rates for development of life stages of P . solenopsis on hibiscus at constant temperatures. (DOCX) [file pone.0075636.s002.docx]

**Table S2. Estimates of non-linear model parameters fitted to the mean rates for development of life stages of *P. solenopsis* on hibiscus at constant temperatures**

| Non-linear model | Parameter |  | Female | | Male | | | Cumulative | | Generation (crawler to crawler) |
| --- | --- | --- | --- | --- | --- | --- | --- | --- | --- | --- |
|  |  | I instar | II instar | III instar | II instar | Prepupa | Pupa | Female | Male |  |
| Lactin-2 | λ | -1.16 ± 0.05 | -1.16 ± 0.03 | -1.11 ± 0.02 | -1.17 ± 0.03 | -1.12 ± 0.02 | -1.11 ± 0.03 | -1.05 ± 0.01 | -1.05 ± 0.01 | -1.04 ± 0.01 |
|  | ρ ± SE (×10^2^) | 1.16 ± 0.17 | 1.23 ± 0.1 | 0.95 ± 0.1 | 1.32 ± 0.1 | 1.08 ± 0.1 | 1.37 ± 0.1 | 0.43 ± 0.05 | 0.37 ± 0.03 | 0.28 ± 0.04 |
|  | Δ | 2.28 ± 1.21 | 2.39 ± 0.65 | 2.56 ± 0.63 | 2.76 ± 0.58 | 2.42 ± 0.37 | 2.05 ± 0.57 | 2.27 ± 0.81 | 2.23 ± 0.57 | 2.24 ± 0.81 |
| Briere-1 | *a* ± SE (×10^4^) | 1.62 ± 0.21 | 1.71 ± 0.15 | 1.18 ± 0.10 | 1.77 ± 0.12 | 1.42 ± 0.08 | 1.99 ± 0.21 | 0.49 ± 0.05 | 0.43 ± 0.03 | 0.32 ± 0.03 |
| Beta type | *α* | 4.001 ± 0.03 | 4.004 ± 0.02 | 4.007 ± 0.03 | 4.003 ± 0.02 | 4.008 ± 0.02 | 4.015 ± 0.04 | 4.004 ± 0.02 | 4.005 ± 0.02 | 4.002 ± 0.02 |
|  | *β* | 4.83 ± 0.38 | 4.46 ± 0.25 | 4.04 ± 0.26 | 4.33 ± 0.19 | 4.10 ± 0.23 | 3.76 ± 0.35 | 4.44 ± 0.28 | 4.34 ± 0.24 | 4.62 ± 0.25 |
|  | *k* ± SE (×10^3^) | 1.31 ± 0.5 | 2.22 ± 0.59 | 2.71 ± 0.75 | 2.71 ± 0.55 | 2.98 ± 0.72 | 6.82 ± 2.47 | 0.66 ± 0.2 | 0.65 ± 0.17 | 0.35 ± 0.1 |
| SSI | *ρ_ϕ_* | 0.0542 | 0.1675 | 0.1480 | 0.1735 | 0.1747 | 0.2305 | 0.0490 | 0.0493 | 0.0316 |
|  | ΔH_A_ | 19275.2 | 17871.2 | 15584.6 | 18215.9 | 14420.7 | 12899.6 | 17508.6 | 16987.8 | 18200.8 |
|  | ΔH_L_ | -708395.5 | -1278.5 | -7654.4 | -1265.9 | -49960.6 | -68905.4 | -1275.1 | -5995.5 | -1284.5 |
|  | ΔH_H_ | 698685.9 | 90105.2 | 77127.7 | 90090.0 | 66271.7 | 43219.1 | 90080.4 | 82600.3 | 90088.8 |
|  | T_L_^*^ | 12.1 | 11.5 | 10.4 | 11.6 | 10.6 | 8.8 | 11.3 | 11.2 | 11.5 |
|  | T_H_ | 35.1 | 35.7 | 36.0 | 35.3 | 37.1 | 38.6 | 35.7 | 35.9 | 35.6 |

*****Lower developmental threshold values from SSI model shown as final T_L_ values when negative T_L_ estimates were obtained with the default option of optTL= 1 in OptimSSI-Program
