# Supplementary material for: Bioclimatic Thresholds, Thermal Constants and Survival of Mealybug, Phenacoccus solenopsis (Hemiptera: Pseudococcidae) in Response to Constant Temperatures on Hibiscus
Source: PLoS One. 2013 Sep 25;8(9):e75636. doi: 10.1371/journal.pone.0075636 (PMC3783440; doi:10.1371/journal.pone.0075636)
Supplement: Table S3 — Results of t-test for evaluation of lower development thresholds (LDTs) and sum of effective temperatures (SETs) of geographical populations of P . solenopsis . (DOCX) [file pone.0075636.s003.docx]

**Table S3: Results of t-test for evaluation of lower development thresholds (LDTs) and sum of effective temperatures (SETs) of geographical populations of *P. solenopsis***

| Study pair | Life stage | LDT*^a^* | | | SET*^a^* | | |
| --- | --- | --- | --- | --- | --- | --- | --- |
|  |  | df | t | *P* | df | t | *P* |
| This study & Hibiscus [11] | Cumulative female | 8 | 2.70 | 0.0270 | 8 | 1.49 | 0.1744 |
|  | Cumulative male | 7 | 1.01 | 0.3462 | 7 | 1.38 | 0.2113 |
|  | Generation | 5 | 1.95 | 0.1087 | 7 | 1.65 | 0.1421 |
| This study & Pumpkin [27] | Cumulative female | 8 | 3.42 | 0.0091 | 8 | 12.87 | <0.0001 |
|  | Cumulative male | 7 | 3.99 | 0.0053 | 7 | 8.12 | 0.0001 |
|  | Generation | 5 | 2.95 | 0.0319 | 7 | 9.53 | <0.0001 |
| Hibiscus [11] & Pumpkin [27] | Cumulative female | 6 | 6.60 | 0.0002 | 6 | 15.61 | <0.0001 |
|  | Cumulative male | 6 | 4.96 | 0.0025 | 6 | 9.11 | 0.0001 |
|  | Generation | 6 | 5.01 | 0.0024 | 6 | 6.65 | 0.0006 |

*^a^* Significance tested at *α* = 0.01 for cumulative female and male nymphs, and *α* = 0.05 for generation
