# Supplementary material for: Bioclimatic Thresholds, Thermal Constants and Survival of Mealybug, Phenacoccus solenopsis (Hemiptera: Pseudococcidae) in Response to Constant Temperatures on Hibiscus
Source: PLoS One. 2013 Sep 25;8(9):e75636. doi: 10.1371/journal.pone.0075636 (PMC3783440; doi:10.1371/journal.pone.0075636)
Supplement: Table S4 — Evaluation of intrinsic optimum temperature (Tϕ) estimates from SSI model for geographical populations of P . solenopsis reared on different hosts. (DOCX) [file pone.0075636.s004.docx]

**Table S4: Evaluation of intrinsic optimum temperature (T_ϕ_) estimates from SSI model for geographical populations of *P. solenopsis* reared on different hosts**

| Study pair | Interval of difference in 95% CI of T_ϕ_^a^ | | | | | |
| --- | --- | --- | --- | --- | --- | --- |
|  | Cumulative female | | Cumulative male | | Generation | |
|  | LL | UL | LL | UL | LL | UL |
| This study & Cotton [26] | -10.00 | 8.20 | -8.81 | 4.24 | -7.51 | 4.88 |
| This study & Hibiscus [11] | -9.11 | 6.44 | -7.21 | 5.33 | -8.75 | 6.21 |
| This study & Pumpkin [27] | -7.09 | 10.82 | -5.04 | 6.34 | -5.26 | 8.69 |
| Cotton [26] & Hibiscus [11] | -5.05 | 6.22 | -4.46 | 7.13 | -3.24 | 4.13 |
| Cotton [26] & Pumpkin [27] | -4.92 | 9.38 | -3.50 | 9.48 | -4.02 | 7.53 |
| Hibiscus [11] & Pumpkin [27] | -3.40 | 6.90 | -3.33 | 6.47 | -3.86 | 7.50 |

^a^LL and UL are lower and upper limits of the 95% confidence interval (CI) of the difference between two groups of bootstrap replications of T_ϕ_. All the intervals contain 0, and thus T_ϕ_ estimate is constant across populations [25].
